# Comparison of COVID-19 Pandemic Dynamics in Asian Countries with Statistical Modeling

**DOI:** 10.1155/2020/4296806

**Published:** 2020-06-28

**Authors:** Min Zuo, Saima K. Khosa, Zubair Ahmad, Zahra Almaspoor

**Affiliations:** ^1^School of Management, China University of Mining and Technology, Xuzhou City, Jiangsu Province, China; ^2^Department of Statistics, Bahauddin Zakariya University, Multan, Pakistan; ^3^Department of Statistics, Yazd University, P.O. Box 89175-741, Yazd, Iran

## Abstract

In the current scenario, the outbreak of a pandemic disease COVID-19 is of great interest. A broad statistical analysis of this event is still to come, but it is immediately needed to evaluate the disease dynamics in order to arrange the appropriate quarantine activities, to estimate the required number of places in hospitals, the level of individual protection, the rate of isolation of infected persons, and among others. In this article, we provide a convenient method of data comparison that can be helpful for both the governmental and private organizations. Up to date, facts and figures of the total the confirmed cases, daily confirmed cases, total deaths, and daily deaths that have been reported in the Asian countries are provided. Furthermore, a statistical model is suggested to provide a best description of the COVID-19 total death data in the Asian countries.

## 1. Introduction

Coronavirus disease (COVID-19) is an infectious disease caused by a newly discovered coronavirus. The name “coronavirus” is derived from the Latin corona, meaning “crown” or “wreath.” The name refers to the characteristic appearance of virions (the infective form of the virus) by electron microscopy.

Coronaviruses were first discovered in the 1930s when an acute respiratory infection of domesticated chickens was caused by infectious bronchitis virus (IBV). Later, in the 1940s, two more animal coronaviruses, mouse hepatitis virus (MHV) and transmissible gastroenteritis virus (TGEV), were isolated [1]. For the first time, human coronaviruses were discovered in the 1960s [2]. The earliest ones studied were from human embryonic tracheal organ cultures obtained from the respiratory tract of an adult with a common cold, which were later named human coronavirus 229E and human coronavirus OC43 [3]. Other human coronaviruses have since been identified, including SARS-CoV in 2003, HCoV NL63 in 2004, HKU1 in 2005, and MERS-CoV in 2012 ([4]). Most of these have involved serious respiratory tract infections.

Recently, a new type of coronaviruses observed in a place called Wuhan city of China, which has a well-known seafood wholesale market, where a large number of people come to sell or buy live seafood. On 31 December 2019, the Wuhan Municipal Health Commission (WMHC) reported a bunch of 27 pneumonia cases of unknown aetiology. Later, on 11 January 2020, the World Health Organization (WHO) named this novel coronavirus as SARS-CoV-2, the virus causing COVID-19, see [5].

The SARS-CoV, MERS-CoV, and COVID-2019 viruses are highly pathogenic Betacoronaviruses and responsible for causing a respiratory and gastrointestinal syndrome. The average incubation period for coronavirus infection is 5 days, with an interval that can reach up to 16 days. The transmissibility of patients infected with SARSCoV is on average 7 days after the onset of symptoms. However, preliminary data from COVID-19 suggests that transmission may occur, even without the appearance of signs and symptoms; see https://en.wikipedia.org/wiki/Coronavirus_disease_2019.

## 2. Detail and Comparison of COVID-19 Cases in the Asian Countries

Asia is one of the most affected region due to COVID-19. In this section, we provide the detailed information and comparison of the total cases, total deaths, total recovered, and active cases in Asian countries. The detail description of the total cases, total deaths, total recovered, and active cases in the Asian countries up to 8th April 2020, are provided in Tables 1 and 2. For details, we refer to https://www.worldometers.info/coronavirus/#countries. Note that the graphical visualization of total cases, total deaths, total recovered, and active cases of the COVID-19 of the Asian countries are displayed in Appendix A. We provide a very simple method for comparison which is not only limited to the Asian countries but it can also be applied for every country to analyze the impact of the disease.

## 3. Proposed Family of Statistical Models

In the practice of big data sciences, particularly in statistical theory, there has be an increased interest in defining new statistical models or new families of statistical models to provide a better description of the problems under consideration; see [6, 7]. For more details, we refer to [8].

Often, adding extra parameter(s) gives more flexibility to a class of distribution functions, improves the characteristics, and provides better fits to the real-life data than the other modified models. But, unfortunately, on the other hand, the reparametrization problem arises. To avoid such problems and provide a better description of real phenomena of nature, we further carry this branch of statistical theory and propose a new class of statistical models. The proposed class of distributions may be called a new flexible extended-*X* (NFE-*X*) class of distributions.

Let *p*(*t*) be the density of a random variable *T* ∈ [*a*_1_, *a*_2_] for −∞≤*a*_1_ < *a*_2_ < ∞ and let *K*[*F*(*x*; *ξ*)] be a function of *F*(*x*; *ξ*) of a random variable *X*. The cumulative distribution function (cdf) of the T-*X* family of distributions [9] is given by

(1) $$\begin{array}{c} G\left(x\right)=\int_{a_{1}}^{K\left[F\left(x;\xi \right)\right]}p\left(t\right)dt,x\in \mathbb{R}, \end{array}$$

where *K*[*F*(*x*; *ξ*)] fulfills some certain conditions, see [9]. The density function corresponding to (1) is

(2) $$\begin{array}{c} g\left(x\right)=\left\{\frac{\partial }{\partial x}K\left[F\left(x;\xi \right)\right]\right\}p\left\{K\left[F\left(x;\xi \right)\right]\right\},\;x\in \mathbb{R}. \end{array}$$

If *p*(*t*) = 1 − *e*^−*t*^, *t* ≥ 0, and setting *K*[*F*(*x*; *ξ*)] = −log((1 − *F*(*x*; *ξ*)^2^)/(*e*^*F*(*x*; *ξ*)^2^^)) in (1), we get the cdf of the proposed class of distributions. The random variable *X* is said to have a NFE-*X* class of distributions, if the cumulative distribution function (cdf) of *X*, denoted by *G*(*x*; *ξ*) is given by

(3) $$\begin{array}{c} G\left(x;\xi \right)=1-\left(\frac{1-F{\left(x;\xi \right)}^{2}}{e^{F{\left(x;\xi \right)}^{2}}}\right),\;x,\xi \in \mathbb{R}. \end{array}$$

The density function corresponding to (3) is

(4) $$\begin{array}{c} g\left(x;\xi \right)=\frac{2f\left(x;\xi \right)F\left(x;\xi \right)}{e^{F{\left(x;\xi \right)}^{2}}}\left\{2-F{\left(x;\xi \right)}^{2}\right\},\;x\in \mathbb{R}. \end{array}$$

One of the most prominent motivations of the proposed approach is to introduce a new class of distributions without adding additional parameter results in avoiding rescaling problems. The next section offers, a special submodel of the proposed class called a new flexible extended-Weibull (NFE-Weibull) distribution and investigates the graphical behaviour of its density function.

## 4. Submodel Description

This section offers a special submodel of the NFE-*X* class of distributions. Let *F*(*x*; *ξ*) be the distribution function of the Weibull model given by *F*(*x*; *ξ*) = 1 − *e*^−*ηx*^*θ*^^, *x* ≥ 0, *η*, *θ* > 0, where *ξ* = (*η*, *θ*). Then, the cdf of the NFE-Weibull has the expression given by

(5) $$\begin{array}{c} G\left(x;\xi \right)=1-\left(\frac{1-{\left(1-e^{-\eta x^{\theta }}\right)}^{2}}{e^{{\left(1-e^{-\eta x^{\theta }}\right)}^{2}}}\right),\;x\geq 0,\eta ,\theta >0, \end{array}$$

with density function

(6) $$\begin{array}{c} g\left(x;\xi \right)=\frac{2\eta \theta x^{\theta -1}e^{-\eta x^{\theta }}\left(1-e^{-\eta x^{\theta }}\right)}{e^{{\left(1-e^{-\eta x^{\theta }}\right)}^{2}}}\left(2-{\left(1-e^{-\eta x^{\theta }}\right)}^{2}\right),\;x>0. \end{array}$$

**Figure 1:**
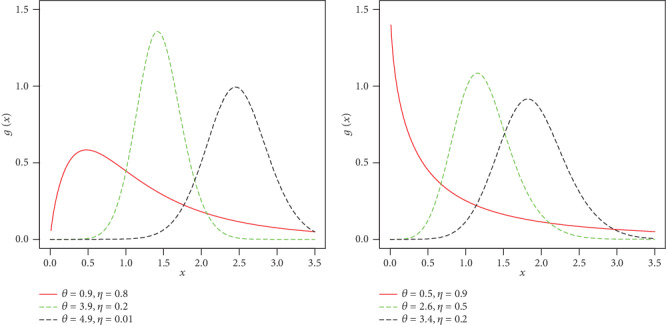
Different plots for the density function of the NFE-Weibull distribution.

For different values of the model parameters, plots of the density function of the NFE-Weibull model are sketched in Figure 1.

## 5. Mathematical Properties

In this section, some mathematical and statistical properties of the NFE-Weibull distribution derived are discussed.

### 5.1. Quantile Function

The quantile function of the NFE-*X* family is the function *Q*(*u*; *ξ*) that satisfies the nonlinear equation

(7) $$\begin{array}{c} Q\left(G\left(u;\xi \right);\xi \right)=u,\;u\in \left(0,1\right). \end{array}$$

By using (3) in (7), after some algebraic manipulation, we get

(8) $$\begin{array}{c} Q\left(G\left(u;\xi \right);\xi \right)=G^{-1}\left(u\right)=F^{-1}\left(u\right), \end{array}$$

where *t* is the solution of log(1 − *u*) + *F*(*x*; *ξ*)^2^ − log(1 − *F*(*x*; *ξ*)^2^).

### 5.2. Moments

Here, we derive some of the moments for the NFE-*X* family. For the sake of simplicity we omit the dependency of *g*(*x*; *ξ*) and *G*(*x*; *ξ*) on the parameter vector *ξ*. The density (4) can be represented as follows:

(9) $$\begin{array}{c} g\left(x\right)=2\frac{f\left(x;\xi \right)F\left(x;\xi \right)}{e^{F{\left(x;\xi \right)}^{2}}}\left\{2-F{\left(x;\xi \right)}^{2}\right\},\\ g\left(x\right)=\overset{1}{\underset{i=0}{\sum }}{\left(-1\right)}^{i}2^{\left(\left(4-i\right)/2\right)}\left(\begin{array}{c} 1\\ i \end{array}\right)\frac{f\left(x;\xi \right)F{\left(x;\xi \right)}^{2i+1}}{e^{F{\left(x;\xi \right)}^{\theta }}},\\ g\left(x\right)=\overset{1}{\underset{i=0}{\sum }}\overset{\infty }{\underset{j=0}{\sum }}\frac{{\left(-1\right)}^{\left(i+j\right)}2^{\left(\left(4-i\right)/2\right)}}{j!}\left(\begin{array}{c} 1\\ i \end{array}\right)f\left(x;\xi \right)F{\left(x;\xi \right)}^{2\left(i+j\right)+1}, \end{array}$$

Using the pdf and cdf of the Weibull distribution in (9), we get

(10) $$\begin{array}{c} g\left(x\right)=\overset{1}{\underset{i=0}{\sum }}\overset{\infty }{\underset{j=0}{\sum }}\overset{2\left(i+j\right)+1}{\underset{k=0}{\sum }}\frac{{\left(-1\right)}^{\left(i+j+k\right)}2^{\left(\left(4-i\right)/2\right)}}{j!}\left(\begin{array}{c} 1\\ i \end{array}\right)\left(\begin{array}{c} 2\left(i+j\right)+1\\ k \end{array}\right)\tau_{k,\eta ,\theta }, \end{array}$$

where *τ*_*k*,*η*,*θ*_ = *ηθx*^*θ*−1^*e*^−*η*(*k* + 1)*x*^*θ*^^. For any positive integer *r*, the *r*^th^ moment of the NFE-Weibull distribution is given by

(11) $$\begin{array}{c} \mu_{r}^{\prime }=E\left(X^{r}\right)=\int_{0}^{\infty }x^{r}g\left(x\right)dx. \end{array}$$

On using (10) in (11), we get the *r*^th^ moment of the NFE-Weibull distribution.

**Figure 2:**
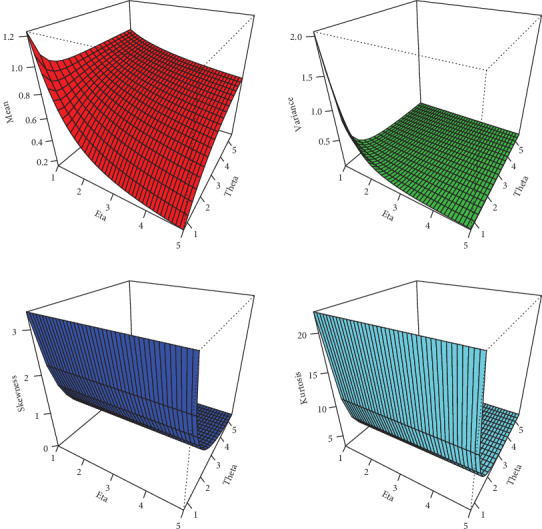
Plots of mean, variance, skewness, and kurtosis of the NFE-Weibull distribution.

**Figure 3:**
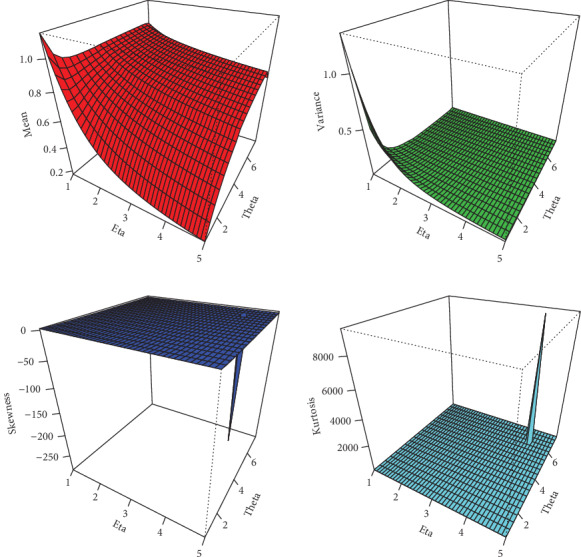
Plots of mean, variance, skewness, and kurtosis of the NFE-Weibull distribution.

For *r* = 1, 2, 3, 4 we get the first four moments of the NFE-*X* distributions. The effects of the shape parameters on the skewness and kurtosis can be detected on the moments. Based on moments, we obtain skewness and kurtosis measures of the NFE-Weibull distribution. The skewness of the NFE-Weibull distribution is obtained as using the following expression:

(12) $$\begin{array}{c} \text{Skewness}=\frac{\mu_{3}}{\mu_{2}^{3/2}}, \end{array}$$

where *μ*_2_ and *μ*_3_ are the second and third moments of the random variable *X* with pdf (6). Furthermore, the kurtosis of *X* is derived as

(13) $$\begin{array}{c} \text{Kurtosis}=\frac{\mu_{4}}{\mu_{2}^{2}}, \end{array}$$

where *μ*_4_ is the fourth moment of *X*. These measures are less sensitive to outliers. Plots for the mean, variance, skewness, and kurtosis of the NFE-Weibull distribution are displayed in Figures 2 and 3.

### 5.3. On Other Means and Moments

With *t* > 0, the following result proposes an expansion of the primitive

(14) $$\begin{array}{c} \int_{0}^{t}x^{r}g\left(x\right)dx=\overset{1}{\underset{i=0}{\sum }}\overset{\infty }{\underset{j=0}{\sum }}\overset{2\left(i+j\right)+1}{\underset{k=0}{\sum }}\frac{{\left(-1\right)}^{\left(i+j+k\right)}2^{\left(\left(4-i\right)/2\right)}}{j!}\left(\begin{array}{c} 1\\ i \end{array}\right)\left(\begin{array}{c} 2\left(i+j\right)+1\\ k \end{array}\right)\tau_{r,k,\eta ,\theta }, \end{array}$$

where *τ*_*r*,*k*,*η*,*θ*_ = ∫_0_^*t*^*ηθx*^*r*+*θ*−1^*e*^−*η*(*k* + 1)*x*^*θ*^^.

Several crucial conditional moments can be obtained using the integral ∫_0_^*t*^*x*^*r*^*g*(*x*)*dx* for various values of *r*. The most useful of them are presented below. For any *t* > 0,

(i) The *r*^th^ conditional moments of *X* is given by,
  (15) $$\begin{array}{c} E\left(X^{r}\;|\;X>t\right)=\frac{1}{1-G\left(t\right)}\int_{t}^{+\infty }x^{r}g\left(x\right)dx=\frac{1}{1-G\left(t\right)}\left(E\left(X^{r}\right)+\int_{0}^{t}x^{r}g\left(x\right)dx\right) \end{array}$$
(ii) The *r*^th^ reversed moments of *X* is given by
  (16) $$\begin{array}{c} E\left(X^{r}\;|\;X\leq t\right)=\frac{1}{G\left(t\right)}\int_{0}^{t}x^{r}g\left(x\right)dx \end{array}$$
(iii) The mean deviations of *X* about the mean, say *μ* is given by
  (17) $$\begin{array}{c} \delta =E\left(\left|X-\mu \right|\right)=2\mu G\left(\mu \right)-2\int_{0}^{\mu }xg\left(x\right)dx \end{array}$$
  where *μ* = *E*(*X*).
(iv) The mean deviations of *X* about the median, say *M* is given by
  (18) $$\begin{array}{c} \tau =E\left(\left|X-M\right|\right)=\mu -2\int_{0}^{M}xg\left(x\right)dx \end{array}$$
 The residual life parameters can be also determined using *E*(*X*^*r*^) and ∫_0_^*t*^*x*^*r*^*g*(*x*; *θ*, *η*)*dx* for several values of *r*. In particular,
(v) The mean residual life is defined as
  (19) $$\begin{array}{c} K\left(t\right)=E\left(X-t\;|\;X>t\right)=\frac{1}{S\left(t\right)}\left(E\left(X\right)-\int_{0}^{t}xg\left(x\right)dx\right)-t \end{array}$$
  and the variance residual life is given by
  (20) $$\begin{array}{c} V\left(t\right)=Var\left(X-t\;|\;X>t\right)=\frac{1}{S\left(t\right)}\left(E\left(X^{2}\right)-\int_{0}^{t}x^{2}g\left(x\right)dx\right)-t^{2}-2tK\left(t\right)-{\left[K\left(t\right)\right]}^{2} \end{array}$$
(vi) The mean reversed residual life is defined as
  (21) $$\begin{array}{c} L\left(t\right)=E\left(t-X\;|\;X\leq t\right)=t-\frac{1}{G\left(t\right)}\int_{0}^{t}xg\left(x\right)dx \end{array}$$
  and the variance reversed residual life is defined as
  (22) $$\begin{array}{c} W\left(t\right)=Var\left(t-X\;|\;X\leq t\right)=\frac{1}{G\left(t\right)}\int_{0}^{t}x^{2}g\left(x\right)dx+2tL\left(t\right)-{\left[L\left(t\right)\right]}^{2}-t^{2} \end{array}$$

## 6. Maximum Likelihood Estimation and Monte Carlo Simulation

The section deals with the estimation of the model parameters and Monte Carlo simulation to assess the performance of the estimators.

### 6.1. Maximum Likelihood Estimation

The maximum likelihood estimation procedure is the commonly employed method of estimating the model parameters. The estimators that are obtained based on this procedure enjoy desirable asymptotic properties, and therefore, they are often utilized to obtain confidence intervals (CI) and test of statistical hypotheses. Suppose that *x*_1_, *x*_2_, ⋯, *x*_*n*_ be the observed values of a random sample of size *n* obtained from (4). The corresponding log-likelihood function can be expressed as

(23) $$\begin{array}{c} \ell \left(\xi \right)=n\log2+\overset{n}{\underset{i=0}{\sum }}\log f\left(x_{i};\xi \right)+\overset{n}{\underset{i=0}{\sum }}\log F\left(x_{i};\xi \right)-\overset{n}{\underset{i=0}{\sum }}F{\left(x_{i};\xi \right)}^{2}+\overset{n}{\underset{i=0}{\sum }}\log\left[2-F{\left(x_{i};\xi \right)}^{2}\right]. \end{array}$$

The log-likelihood function can be maximized either directly or by solving the nonlinear likelihood function obtained by differentiating. The first-order partial derivative of the log-likelihood function with respect to *ξ* is given by

(24) $$\begin{array}{c} \frac{\partial }{\partial \xi }\ell \left(\xi \right)=\overset{n}{\underset{i=0}{\sum }}\frac{\partial f\left(x_{i};\xi \right)/\partial \xi }{f\left(x_{i};\xi \right)}+\overset{n}{\underset{i=0}{\sum }}\frac{\partial F\left(x_{i};\xi \right)/\partial \xi }{F\left(x_{i};\xi \right)}-2\overset{n}{\underset{i=0}{\sum }}\frac{F\left(x_{i};\xi \right)\partial F\left(x_{i};\xi \right)}{\partial \xi }-2\overset{n}{\underset{i=0}{\sum }}\frac{F\left(x_{i};\xi \right)\partial F\left(x_{i};\xi \right)/\partial \xi }{2-F{\left(x_{i};\xi \right)}^{2}}. \end{array}$$

Setting (*∂*/*∂ξ*)*ℓ*(*ξ*) equal to zero and solving numerically yields the maximum likelihood estimators (MLEs) of *ξ* = (*θ*, *η*). An optimization software such as the R function *optim* or *nlminb* can be used to find $\hat{\xi }$ that minimizes the negative log-likelihood function (i.e., maximizes the log-likelihood function). Although the specification of the derivatives is optional in these R functions, fast and rapid convergence may be achieved if the expressions for the negative log-likelihood function are provided. In our implementation (R codes are given in Appendix B), we use optim() R-function with the argument method = “SANN” to obtain the MLEs.

### 6.2. Monte Carlo Simulation

A numerical investigation is established to examine the behaviour of MLEs for the NFE-Weibull model. For different sample sizes, measures like biases, absolute biases, and mean square errors (MSEs) are calculated to evaluate the performance of the estimators.

- We generate 500 from NFE-Weibull distribution of sizes; *n* = 25, 50, ⋯, 500
- An optimization algorithm requires a set of initial values for the parameters. Certain values of the model parameters (*θ*, *η*) are chosen as Set 1 : *θ* = 0.5, *η* = 1; Set 2 : *θ* = 1.2, *η* = 1; and Set 3 : *θ* = 0.8, *η* = 1.2
- MLEs of the parameters *θ* and *η* are calculated for each *n* and for all sets
- Calculate the biases, absolute biases, and MSE for each *n*

**Figure 4:**
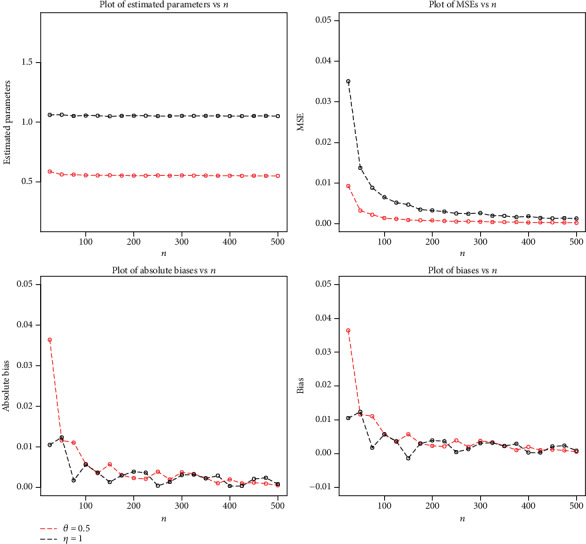
Plots of MLEs, MSEs, biases, and absolute biases for *θ* = 0.5 and *η* = 1.

**Figure 5:**
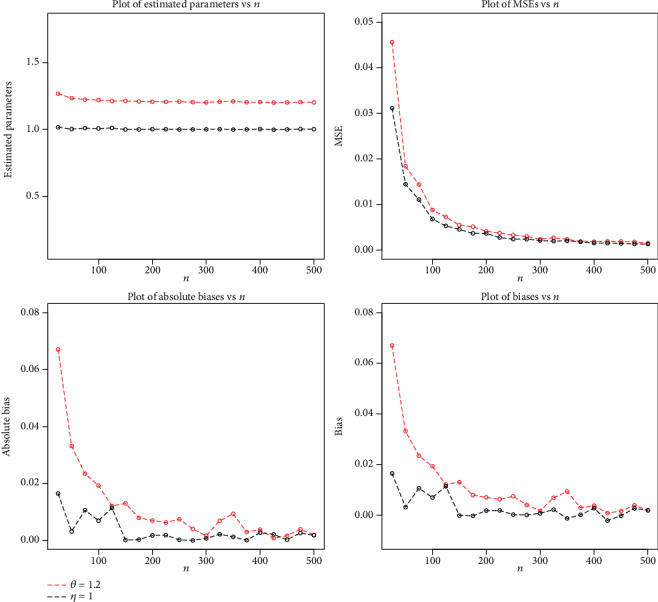
Plots of MLEs, MSEs, biases, and absolute biases for *θ* = 1.2 and *η* = 1.

**Figure 6:**
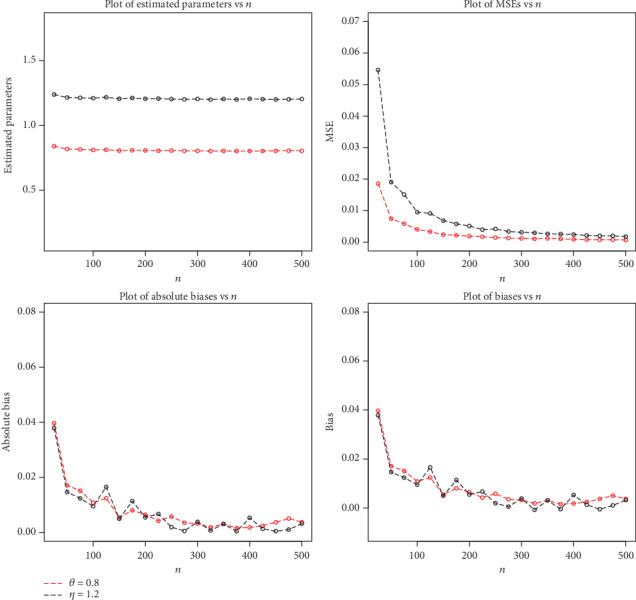
Plots of MLEs, MSEs, biases, and absolute biases for *θ* = 0.8 and *η* = 1.2.

The simulation results are displayed in Figures 4–6.

## 7. Modeling COVID-19 Total Deaths of the Asian Countries

We mentioned earlier that a broad statistical analysis of the events that occurred due to COVID-19 is still to come. But, now it is immediately needed to propose a suitable model to provide a better description of the COVID-19 total death data to estimate the required number of places in hospitals, the level of individual protection, the rate of isolation of infected persons, etc. In this section, we model the COVID-19 total deaths that have occurred in the Asian countries up to April 8, 2020. The NFE-Weibull distribution applied to this dataset in comparison with the other well-known distributions such as the two-parameter Weibull, three-parameter Marshall-Olkin Weibull (MOW), and exponentiated Weibull (EW) distributions. It is important to emphasize that the EW distribution is a popular model for analyzing data in the applied areas, particularly in medical sciences, see [10]. The MOW distribution is another nonnested model and offers the characteristics of the Weibull and gamma distributions, see [11]. The cdfs of the competing distributions are as follows:

(1) Weibull distribution
  (25) $$\begin{array}{c} G\left(x;\theta ,\eta \right)=1-e^{-\eta x^{\theta }},\;x\geq 0,\theta ,\eta >0 \end{array}$$
(2) EW distribution
  (26) $$\begin{array}{c} G\left(x;a,\theta ,\eta \right)={\left(1-e^{-\eta x^{\theta }}\right)}^{a},\;x\geq 0,a,\theta ,\eta >0 \end{array}$$
(3) MOW distribution
  (27) $$\begin{array}{c} G\left(x;\theta ,\eta ,\sigma \right)=\frac{1-e^{-\eta x^{\theta }}}{1-\left(1-\sigma \right)\left(1-e^{-\eta x^{\theta }}\right)},\;x\geq 0,\theta ,\eta ,\sigma >0 \end{array}$$

Selection of an appropriate approximation model is desirable to assign some preference to the alternatives. Therefore, we consider certain analytical measures in order to verify which distribution fits better the considered data. These analytical measures include (i) four discrimination measures such as the Akaike information criterion (AIC), Bayesian information criterion (BIC), Hannan-Quinn information criterion (HQIC), and consistent Akaike information criterion (CAIC) and (ii) three other goodness-of-fit measures including the Anderson Darling (AD) test statistic, Cramer-Von-Messes (CM) test statistic, and Kolmogorov-Smirnov (KS) test statistics with corresponding *p* values. A model with lowest values for these statistics is considered a best candidate model. The formulae for these measures can be explored in [12]

For the COVID-19 total death data of the Asian countries, the estimates with the standard error (in parentheses) of the model parameters are provided in Table 3. The analytical measures of the NFE-Weibull and other considered models are provided in Tables 4 and 5.

**Figure 7:**
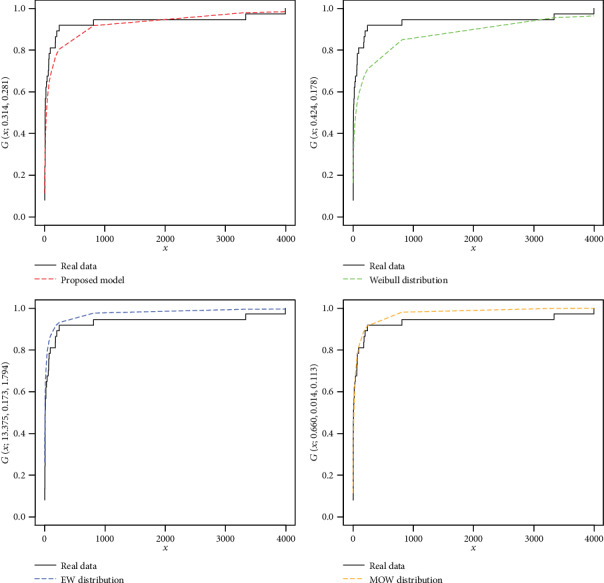
Plots of the estimated cdfs of the NFE-Weibull and other competitive distributions for the COVID-19 total death data of the Asian countries.

**Figure 8:**
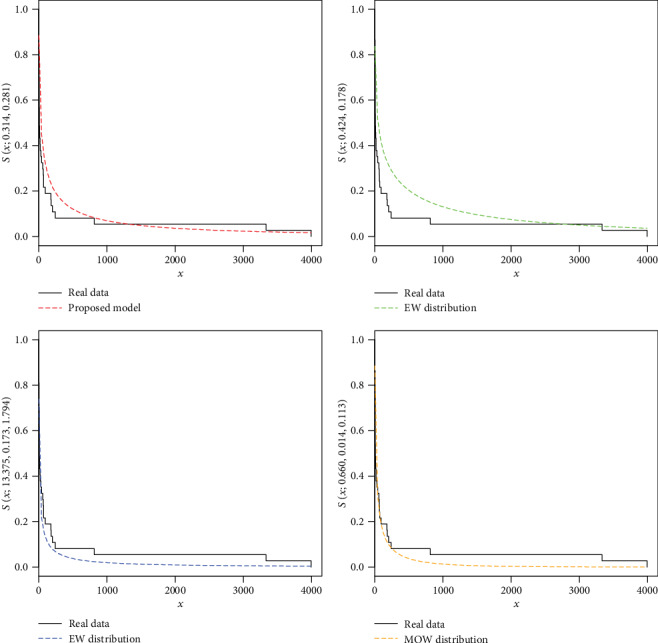
The Kaplan-Meier survival plots of the NFE-Weibull and other competitive distributions for the COVID-19 total death data of the Asian countries.

As we see, the results (Tables 4 and 5) show that the NFE-Weibull distribution has smaller values of the analytical measures and the maximum *p* value reveals that the proposed model provides better fit than the other considered competitors. Hence, the proposed model can be used as a best candidate model for modeling the COVID-19 total death data of the Asian countries. In support of the results provided in Tables 4 and 5, the estimated cdfs of the fitted distributions are plotted in Figure 7, whereas the Kaplan-Meier survival plots of the proposed and other fitted distributions are presented in Figure 8. From Figures 7 and 8, it is clear that the proposed model fit the estimated cdf and survival function very closely than the other competitors.

## 8. Concluding Remarks

The COVID-19 is one among the most deadly viruses that has greatly affected daily life affairs. The government and a number of other organizations should be interested to provide bases for comparison and to provide a better description of the data under consideration to get reliable estimates of the parameters of interest. In this article, a brief comparison of the COVID-19 events such as total cases, total deaths, total recovered, and active cases of the Asian countries are provided. Such clear cut comparison should be helpful to facilitate the COVID-19 affected peoples. Furthermore, a new class of statistical models is introduced. Some mathematical properties of the proposed class are derived. The maximum likelihood estimators of the model parameters are obtained. Finally, a special submodel of the proposed class called a new flexible extended Weibull distribution is studied in detail. The flexibility provided by the proposed model could be very useful in adequately describing the total death data in the Asian countries due to the COVID-19. We observed that the proposed model may provide a close fit to the COVID-19 total death data.

## Figures and Tables

**Figure 9:**
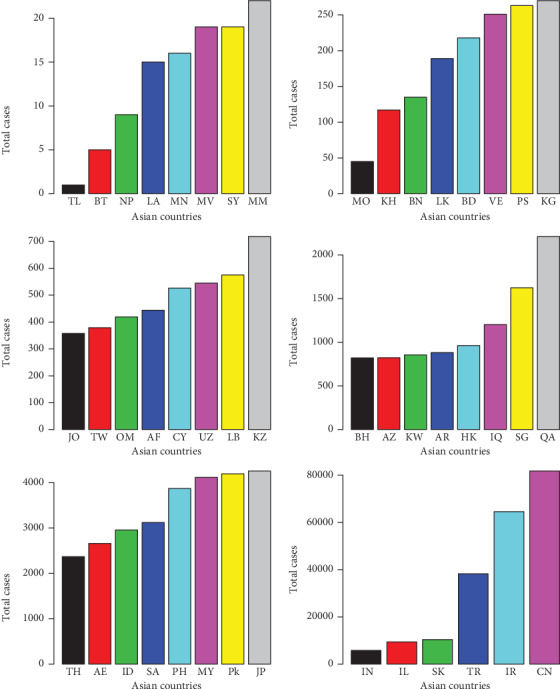
Total cases of the COVID-19 in the Asian countries.

**Figure 10:**
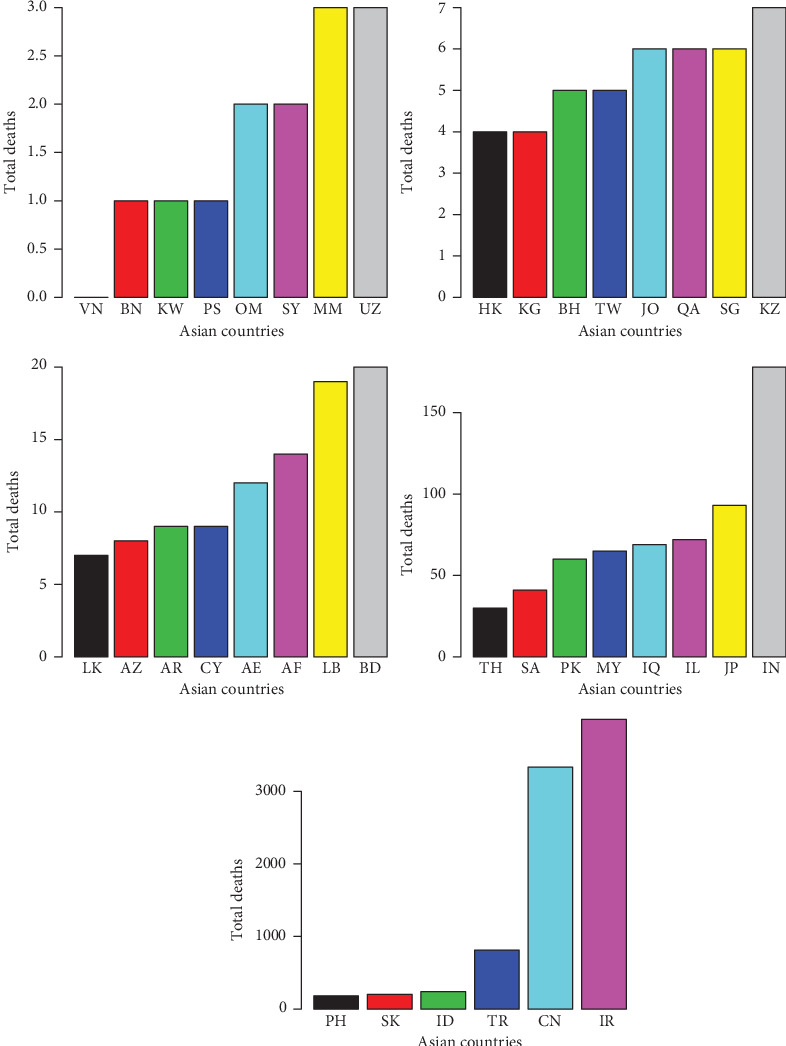
Total deaths due to the COVID-19 in the Asian countries.

**Figure 11:**
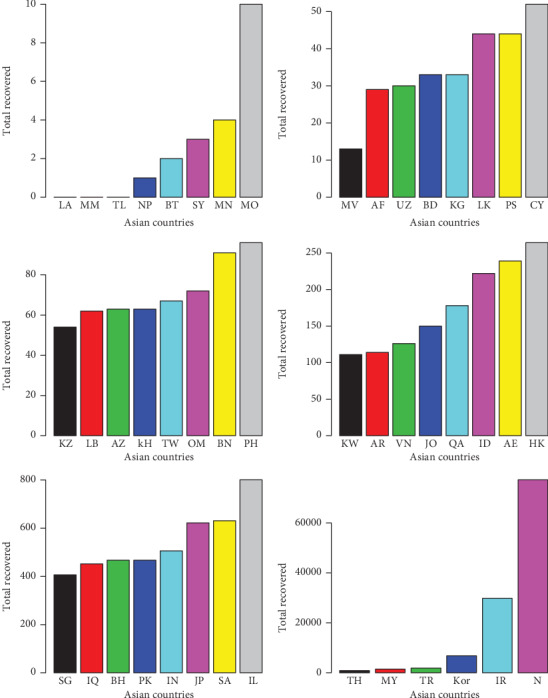
Total recovered cases of COVID-19 in the Asian countries.

**Figure 12:**
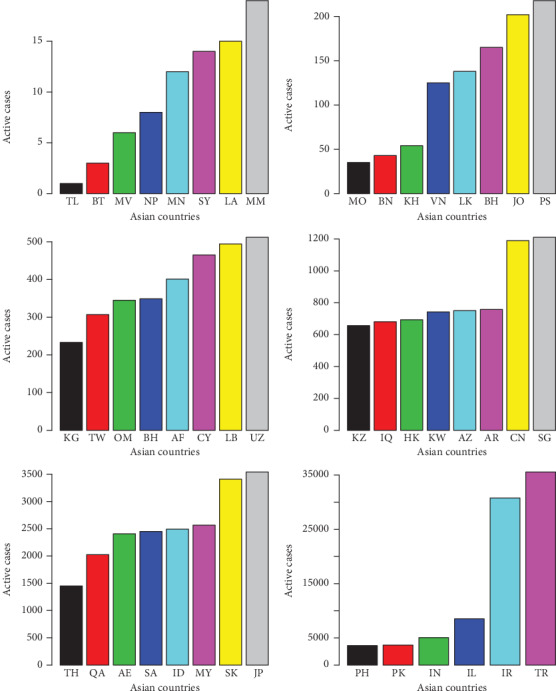
Active cases of the COVID-19 in the Asian countries.

**Table 1:** Detailed report and comparison of COVID-19 cases in the Asian countries.

Countries	Total cases	Total deaths	Total recovered	Active cases
Afghanistan (AF)	444	14	29	401
Azerbaijan (AZ)	822	8	63	751
Bahrain (BH)	821	5	467	349
Bangladesh (BD)	218	20	33	165
Bhutan (BT)	5	0	2	3
Brunei (BN)	135	1	91	43
Cambodia (KH)	117	0	63	54
China (CN)	81802	3333	77279	1190
Cyprus (CY)	526	9	52	465
Hong Kong (HG)	961	4	264	693
India (IN)	5749	178	506	5065
Indonesia (ID)	2956	240	222	2494
Iran (IR)	64586	3993	29812	30781
Iraq (IQ)	1202	69	452	681
Israel (IL)	9409	72	801	8531
Japan (JP)	4257	93	622	3542
Jordan (JO)	358	6	150	202
Kazakhstan (KZ)	718	7	54	657
Kuwait (KW)	855	1	111	743
Kyrgyzstan (KG)	270	4	33	233
Laos (LA)	15	0	0	15
Lebanon (LB)	575	19	62	494
Macao (MO)	45	0	10	35
Malaysia (MY)	4119	65	1487	2567
Maldives (MV)	19	0	13	6
Mongolia (MN)	16	0	4	12
Myanmar (MM)	22	3	0	19
Nepal (NP)	9	0	1	8
Oman (OM)	419	2	72	345
Pakistan (PK)	4196	60	467	3669
Philippines (PH)	3870	182	96	3592
Qatar (QA)	2210	6	178	2026
Saudi Arabia (SA)	3122	41	631	2450

**Table 2:** Detailed report and comparison of COVID-19 cases in the Asian countries.

Countries	Total cases	Total deaths	Total recovered	Active cases
Singapore (SG)	1623	6	406	1211
South Korea (SK)	10384	200	6776	3408
Sri Lanka (LK)	189	7	44	138
Palestine (PS)	263	1	44	218
Syria (SY)	19	2	3	14
Taiwan (TW)	379	5	67	307
Thailand (TH)	2369	30	888	1451
Timor-Leste (TL)	1	0	0	1
Turkey (TR)	38226	812	1846	35568
United Arab Emirates (AE)	2659	12	239	2408
Uzbekistan (UZ)	545	3	30	512
Vietnam (VN)	251	0	126	125

**Table 3:** Estimated parameters along with standard errors (in parenthesis) of the fitted models.

Distribution	*θ*	*η*	*a*	*σ*
NFE-Weibull	0.314 (0.0392)	0.281 (0.0567)		
Weibull	0.424 (0.0484)	0.178 (0.5544)		
MOW	0.660 (0.0442)	0.014 (0.4690)		0.113 (1.8756)
EW	0.173 (0.0323)	1.794 (0.5113)	13.375 (7.858)	

**Table 4:** Discrimination measures of the NFE-Weibull model and other competing models.

Distribution	AIC	BIC	CAIC	HQIC
NFE-Weibull	386.178	389.400	386.531	387.314
Weibull	394.615	397.837	394.968	395.751
MOW	389.535	392.367	388.262	389.238
EW	392.503	395.336	392.231	393.207

**Table 5:** Goodness-of-fit measures of the NFE-Weibull model and other competing models.

Distribution	CM	AD	KS	*p* value
NFE-Weibull	0.188	1.165	0.152	0.353
Weibull	0.284	1.786	0.193	0.276
MOW	0.209	1.206	0.171	0.329
EW	0.232	1.499	0.186	0.303

## Data Availability

The data used to support the findings of this study are included within the article.

## Appendix

### A. Display of the COVID-19 Events

Figures 9–12 offers the graphical display of the COVID-19 events that have taken place in the Asian Countries.

Note: Since the total deaths for the BT, KH, LA, MO, MV, MN, NP, TL, and VN are zero “0”. Therefore, the plotting of the total deaths for these countries are omitted.

### B. R Codes

#### B.1. R Code for Analysis

The following code has been used to calculate the values of the model parameters.

Note: Here, pm is used for proposed model.

library(AdequacyModel)

data=c(14,9,8,5,20,1,3333,9,4,178,240,3993,69,72,93,6,7,1,4, 19,65,3,2,60,182,6,41,6,200,7,1,2,5,30,812,12,3)

sort(data)

data

################# PDF of the proposed model

pdf_pm <- function(par,x)

{

theta=par[1] eta=par[2] theta∗2∗eta∗(x^(theta-1))∗exp(-eta∗x^theta)∗((1-exp(-eta∗x^theta))) ∗(2-((1-exp(-eta∗x^theta))^2))∗(1/(exp((1-exp(-eta∗x^theta))^2)))

}

################# CDF of the proposed model

cdf_pm <- function(par,x)

{

theta=par[1]

eta=par[2]

1-((1-((1-exp(-eta∗x^theta))^2))/(exp((1-exp(-eta∗x^theta))^2)))

}

set.seed(0)

goodness.fit(pdf=pdf_pm, cdf=cdf_pm, starts = c(1,1), data = data, method="SANN", domain=c(0,Inf),mle=NULL)

#### B.2. R Code for Plotting the Estimated Distribution Function.

x=c(14,9,8,5,20,1,3333,9,4,178,240,3993,69,72,93,6,7,1,4, 19,65,3,2,60,182,6,41,6,200,7,1,2,5,30,812,12,3)

sort(x) x

#NAPTW

theta=0.3147927

eta=0.2818076

x<-sort(x)

F1<-ecdf(x)

ecdf<-F1(c(x)) proposedcdf<-1-((1-((1-exp(-eta∗x^theta))^2))/(exp((1-exp(-eta∗x^theta))^2))) plot(x,ecdf,lty=1,lwd=4,type="s",xlab="x",ylab="G(x; 0.3147927, 0.2818076)", ylim=c(0,1),xlim=c(min(x),max(x)),col="black")

par(new=TRUE)

plot(x,proposedcdf,xlab="x",ylab="G(x; 0.3147927, 0.2818076)", ylim=c(0,1),xlim=c(min(x),max(x)),col="red",lty =5, lwd=4,type="l")

par(new=TRUE)

legend(1500, 0.4,c("Real Data","Proposed Model"),col=c(1,2), lty =c(1,5), bty="n", cex=1.2)

#### B.3. R Code for Plotting the Fitted Survival Function.

x=c(14,9,8,5,20,1,3333,9,4,178,240,3993,69,72,93,6,7,1,4, 19,65,3,2,60,182,6,41,6,200,7,1,2,5,30,812,12,3)

summary(x)

library(survival)

delta=rep(1,length(x))

x<-sort(x)

km = survfit(Surv(x,delta)~1)

plot(km,conf.int=FALSE,ylab="S(x; 0.3147927, 0.2818076)",xlab="x", lty =1, col="black",pch=19,lwd=4)

theta=0.3147927

eta=0.2818076

ss <- function(x)

{

((1-((1-exp(-eta∗x^theta))^2))/(exp((1-exp(-eta∗x^theta))^2)))

}

lines(seq(1, 3993,length.out =100),ss(seq(1,3993,length.out =100)), col="red",lty =5,lwd=4) legend(1500, 0.7,c("Real Data","Proposed Model"),col=c(1,2), lty =c(1,5), bty="n", cex=1.2)
